# Hymenoptera Venom Immunotherapy Meets Factitious Disorder

**DOI:** 10.7759/cureus.55769

**Published:** 2024-03-08

**Authors:** Epameinondas Stratopoulos, Angeliki Leonardou, Constantinos Pitsios

**Affiliations:** 1 School of Medicine, National and Kapodistrian University of Athens, Athens, GRC; 2 Psychiatry, Women's Mental Health Clinic, National and Kapodistrian University of Athens, Athens, GRC; 3 Medical School, University of Cyprus, Nicosia, CYP

**Keywords:** placebo mechanism, allergic asthma, venom immunotherapy, allergen-specific immunotherapy (ait), factitious disorder, insect venom allergy

## Abstract

Factitious disorder on self is a psychiatric disorder in which individuals fabricate or induce signs or symptoms of a disease. Factitious anaphylaxis, with symptoms suggestive of a life-threatening allergic reaction, is extremely rare. Several cases of factitious disorder reactions during allergen immunotherapy for airborne allergens have been reported. We report the case of a young female patient who presented factitious anaphylaxis during venom immunotherapy to vespid venom extract. Symptoms of stridor, dyspnea, coughing and loss of consciousness were observed during the built-up phase of venom immunotherapy, mimicking allergic reactions to the venom extracts. Diagnosis of factitious disorder prompted the discontinuation of venom immunotherapy.

## Introduction

Venom immunotherapy (VIT) is an efficacious treatment for both children and adults who have experienced sting anaphylaxis. VIT prevents subsequent allergic sting reactions and has a beneficial impact on disease-specific quality of life [1-4]. The diagnosis of Hymenoptera venom allergy relies on anamnesis of Hymenoptera sting-related anaphylaxis and the detection of IgE-mediated sensitization, using skin tests and serum-specific IgE [5]. Once the culprit insect is identified and the diagnosis is confirmed, VIT can be initiated. 

While VIT is considered a safe treatment, the occurrence of side effects is common, ranging from large local reactions to life-threatening anaphylaxis [2,3,6]. Allergic reactions during VIT must be promptly and appropriately treated to prevent fatality [2]. Risk factors for systemic anaphylactic reactions during VIT include the treatment with honey-bee venom, anamnesis of severe reactions to stings, elevated basal serum tryptase levels or mastocytosis and the build-up phase of VIT [2,3,7].

Anaphylaxis is characterized by classical allergic symptoms (e.g. urticaria, shortness of breath, throat tightness, nausea, cardiovascular collapse), but some atypical symptoms, such as chills, fever or pain, may also occur [8]. Factitious anaphylaxis is mimicking a life-threatening condition that requires immediate medical attention. Medical literature is limited on this topic [9-12]. Factitious anaphylaxis poses a diagnostic and treatment challenge because prompt treatment must precede the final diagnosis. 

‘Factitious disorders’ was an older classification of psychiatric disorders mimicking the whole range of somatic syndromes and diseases. Depending on the contribution of the unconscious in the production of symptoms three different psychiatric entities were distinguished: hypocrisis, factitious disorder (Munchausen syndrome) and hysteria. The newer categorization of the Diagnostic and Statistical Manual of Psychiatry (DSM-5) has broadened the spectrum, destigmatizing the use of the psychiatric terms, however adding a level of confusion to the diagnosis. According to the DSM-5 these cases belong now to the ‘Somatic Symptoms and Related Disorders’ [13]. Factitious disorder (FD) imposed on self (Munchausen syndrome) is one of the seven disorders in this category [13]. It is a psychiatric disorder characterized by fabrication or induction of signs or symptoms of a disease, as well as alteration of laboratory tests. Patients pretend that they are sick and tend to seek treatment, without secondary gains, at different care facilities [13-15]. These patients often constitute a medical dilemma due to their vague and inconsistent symptoms and clinical image, with multiple presentations [14].

Here, an extremely rare case of a young woman with an FD reaction, mimicking anaphylaxis during VIT, is reported.

## Case presentation

A 17-year-old female reported an episode of dizziness, dyspnea and cough a few minutes after being stung by an unidentified insect in her schoolyard. She was immediately treated by the school nurse with the administration of cetirizine by mouth, intramuscular (i.m.) methylprednisolone, and oxygen application. Symptoms remitted in the next half-hour. An emergency anaphylaxis set was prescribed to her.

She was already a patient of our Allergy Office, with a history of allergic rhinitis and asthma. Almost a year before she had successfully completed a three-year-long treatment of grass pollen sublingual immunotherapy (SLIT). No symptoms of respiratory allergy had occurred since the completion of SLIT. She also had a history of a large local reaction episode, after being stung by an insect identified as a honey-bee (stinger removed from the site of the sting).

Allergy tests were performed with Apis melifera, Vespula spp. and Polistes spp. venom extracts and resulted positive for Vespula spp. (intradermal at 0.01μg/ml concentration). A self-remitting cough was noticed during the allergy test procedure. Allergy to Vespula was confirmed in vitro [i3=5.07 U/ml, (ImmunoCAP specific IgE, Thermo Fisher Scientific, Waltham, MA, USA)] and the relevant venom extract for VIT was prescribed. She had a normal value (2.8μg/L) for tryptase (ImmunoCAP tryptase, Thermo Fisher Scientific). VIT following a modified rush protocol was proposed to her parent.

VIT began about a month after allergy testing. Initiating VIT at the 1μg dose in rush protocols is shown to be safe; however, patient’s cough during allergy tests led to the decision of an initial subcutaneous placebo injection, to exclude a vasovagal reaction [15]. Blood pressure (105/65mmHg), heart rate (84/min) and peak expiratory flow rate (PEFR) were measured before the administration of the first (placebo) injection.

Five minutes after the placebo injection the patient started to cough, with a slight decrease of PEFR (from initial 410 to 370 L/min) noticed. Fifteen minutes later, PEFR was maintained at a similar level (390 L/min) and a dose of 1μg (0.1mL of a 10μg/mL Vespula venom solution) was administered. In order to follow a safer pathway, the following doses were lower than the intended ones, so 2μg, 3μg and 5μg were administered, keeping an interval of 30 minutes (Table 1).

**Table 1:** Doses of venom immunotherapy (VIT) and registered reactions Placebo: 0.1ml of 0.9% saline

Day	Concentration of solution	Dose (μg)	Reactions
1	Placebo	0	cough
	10μg/ml	1	
	"	2	
	"	3	
	"	5	cough, increase in blood pressure and heart rate
	100μg/ml	10	
	"	15	
		Total: 36	
2	100μg/ml	15	
	"	20	
	"	25	cough, dyspnea, wheezing, stridor
		Total: 60	
3	100μg/ml	15	cough
	"	20	“feeling unwell”
		Total: 35	
4	Placebo	0	
	100μg/ml	15	
	"	20	cough, stridor, arm-hand paralysis, loss of consciousness
		Total: 35	

The patient started coughing at the dose of 5μg, maintaining a PEFR of 390L/min and raising blood pressure (175/65mmHg) and heart rate (94/min). VIT continued with the administration of 10μg (0.1ml of 100μg/mL solution) and 15μg. Patient had no reaction following the last two doses, so the total of 36μg was administered on Day 1. She was prescribed a budesonide/formoterol inhaler with the instruction to start using it the same night (two puffs twice per day).

On Day 2, after clinical examination, the patient was set under cardiovascular monitoring, with a device (blood pressure, heart rate, oximeter). VIT was continued with the re-administration of the last tolerated dose of 15μg, followed by doses of 20μg and 25μg, with an hour’s interval between each one. Five minutes after the dose of 25μg the patient reported dyspnea, started to cough, to wheeze and presented laryngeal stridor. Although PEFR (380L/min), pulse oximetry (SatO2: 97%) blood pressure (BP: 103/58mmHg) and heart rate (HR: 66/min) were similar to the ones during clinical examination, i.m. epinephrine (300μg) was immediately administered, while hydrocortisone (500mg) and dimentidene (4mg) were also administered intravenously (i.v.). Nebulization was used with the solution of ipratropium bromide and albuterol sulfate (0.5mg+0.3mg) as well as budesonide (0.5mg).

The patient went on presenting stridor and reported signs of anxiety. Epinephrine (300μg i.m.) was repeated 10 minutes later (vital signs remaining the same), but no improvement was noticed regarding dyspnea or stridor, despite the administration of epinephrine, the nebulization and oxygen provided with nasal canula. Finally, 100μg of 1:10 solution of epinephrine was provided i.v., 15 minutes after the second dose. Although the patient reported that she was gradually feeling better the stridor persisted. She was asked to lie in the supine position on the hospital bed in order to “get some rest” and it was noticed that stridor immediately disappeared.

The poor response of respiratory symptoms to high doses of medication, the steady vital signs and the fact that stridor disappeared in the supine position, along with patient’s reaction to the placebo dose of Day 1, raised suspicions of symptoms’ falsification by the patient. However, continuing VIT was a matter of ethics, since VIT is highly recommended for patients who have experienced a systemic reaction of Hymenoptera venom allergy. The situation was discussed with her mother, revealing that she had been visiting a psychologist over the last months. It was decided to explain to the patient herself that her reaction was considered non-immunologically mediated and to offer her the choice of continuing or stopping VIT. She eagerly replied that she wanted to complete the build-up phase of VIT. She was instructed to adhere to the use of her budesonide/formoterol inhaler and confirmed its usage.

Two days later, on Day 3 of VIT, the patient received an injection of 15μg of venom solution. Cough appeared once again, a few minutes after the injection and remitted slightly with nebulization (ipratropium and albuterol) and the administration of hydrocortisone (500mg) i.v. A second dose of 20μg venom solution was administered, 30 minutes later, but the patient reported feeling unwell, without any change in vital signs. She remained under medical surveillance for an hour and VIT proceeding was postponed for the next day.

During Day 4 of VIT, an initial placebo dose was administered, followed by the same doses of Day 3; 15μg and 30 minutes later the dose of 20μg (Table 1). Five minutes after the latter dose she presented stridor, coughing, arm-hand paralysis and loss of consciousness. Vital signs were kept normal (BP: 91/58mmHg, HR: 72/min, SatO2: 98%), not confirming anaphylactic shock. Epinephrine (300μg i.m.) was administered twice (with an interval of 10 minutes), with nebulization and oxygen. The patient started to communicate soon after the first injection of epinephrine and paralysis symptoms improved too. This latter incidence, which was impossible to attribute to any allergic reaction, proved to us the self-induced character of the symptoms. It was decided to terminate VIT and the parents were advised that the patient should consult a psychiatrist.

## Discussion

Differentiating factitious anaphylaxis from true anaphylaxis is extremely difficult when symptoms like stridor, dyspnea with cough, or fainting occur. Clinical clues that helped in the diagnostic differentiation were alleviation of stridor in the supine position (instead of exacerbation) and the minimal response of cough and dyspnea to bronchodilation and epinephrine administration. Loss of consciousness during anaphylactic shock is an emergency related to a mean blood pressure lower than 65mmHg, not observed in our case. On the contrary arm-hand paralysis was a sign of conversion disorder.

In our case report the young female patient presented with multiple episodes of reactions during VIT. Each episode comprised dyspnea, cough, wheeze and stridor suggesting an asthmatic crisis and laryngeal edema. There is no data supporting more frequent or more severe VIT-related side effects in asthmatics and only partially controlled or uncontrolled asthma respectively consists of a relative or an absolute contraindication to VIT [16,17]. In our case, an exacerbation of asthma symptoms was suspected, due to her anamnesis. It has to be underlined that it was January and the grass pollen period had not started.

She also had an episode of paralysis resembling faint, mimicking anaphylaxis. Factitious anaphylaxis can present as a life-threatening condition that requires immediate medical attention [10]. When symptoms appeared the emergency was to treat them considering the reaction as allergic. Even if there were clear signs that it was an FD reaction, it was impossible to risk not treating the symptoms. Measurement of serum tryptase taken during the reaction could have been helpful to exclude anaphylaxis.

Allergen immunotherapy (AIT) is a broader term that includes VIT, as well as immunotherapy for airborne allergens (e.g. SLIT) and food induction intolerance. It is strongly advised that subcutaneous AIT should be performed in an allergy office/clinic supplied with the appropriate medical equipment and administered by specialists, well-trained to recognize and treat a systemic reaction promptly and efficiently [18]. Until proven otherwise, each reaction during AIT must be treated as an IgE-mediated reaction with appropriate measures to counteract anaphylaxis [19]. These guidelines were followed in our case.

Reactions to placebo in AIT-treated patients are uncommon. In a large series of patients treated with VIT there was no reaction during placebo administration, nor to the initial venom dose of 1μg [19]. Differential diagnosis between an allergic and an FD reaction is difficult, especially when acute urticaria (a common symptom during anaphylaxis) is missing. Besides the reaction to placebo, other clues leading to the diagnosis of FD were the stable vital signs, the protracted coughing and stridor with limited response to repeated doses of epinephrine and nebulized therapy, the stop of stridor upon the supine position and finally the presence of temporary arm-hand paralysis.

To our best knowledge, no case report of factitious anaphylaxis due to VIT has been published before. A case report of Munchausen syndrome mimicking Hymenoptera venom anaphylaxis after a field sting has been retrieved from the literature [20]. Regarding the occurrence of Munchausen syndrome during AIT to aeroallergens, a published case series of six confirmed and four probable cases have been published [18]. On the other hand, self-induced laryngeal stridor has more often been reported as an FD symptom, in patients inculpating foods or drugs [9-12].

Our differential diagnosis also included the Conversion Disorder (Functional Neurological Symptom Disorder), which could have explained the latter symptom of paralysis, but could not explain the cough and the laryngeal stridor, as well as the Psychological Factors Affecting Other Medical Conditions. This diagnosis could be based on the temporal relation of the symptoms with VIT, but it could not explain the absence of abnormal physical findings during the reactions, the abrupt stop of stridor in the supine position, the eagerness of the patient to come repeatedly to the hospital, despite the intense difficulties that she presented, the non-immunologically related paralysis, as well as its remission.

The diagnosis of Somatic Symptoms and Related Disorders, and specifically of FD imposed on self, present a challenge as physical symptoms are largely indistinguishable between endogenous and exogenous forms of the condition. It is important to distinguish these reactions from their immunologic counterparts.

Munchausen syndrome carries significant morbidity and mortality. The treatment of Munchausen syndrome is difficult, and it requires the help of a psychiatrist. Regarding our young patient, VIT was discontinued and psychiatric consultation was advised. Psychiatric referral may be indicated in an attempt to improve the patient’s general psychiatric status and as a result the patient’s response to the allergic disease and its treatment.

## Conclusions

FD should be considered in the differential diagnosis of an allergic emergency due to VIT. Our case report confirms the mandatory use of the clinical exam proceeding immunotherapy, the usefulness of vital sign monitoring in cases presenting numerous reactions during AIT and also the fact that placebo can be of use in various diagnostic issues in Allergology.
